# Would you like fries with that? Investigating fast‐food outlet availability near schools in Perth, Western Australia

**DOI:** 10.1002/hpja.682

**Published:** 2022-12-04

**Authors:** Gina S. A. Trapp, Paula Hooper, Wesley Billingham, Lukar Thornton, Ainslie Sartori, Kelly Kennington, Amanda Devine, Stephanie Godrich, Ros Sambell, Justine Howard, Alexia Bivoltsis

**Affiliations:** ^1^ Telethon Kids Institute Perth Children's Hospital Nedlands WA Australia; ^2^ School of Population and Global Health The University of Western Australia Nedlands WA Australia; ^3^ The Australian Urban Design Research Centre (AUDRC), School of Design The University of Western Australia Nedlands WA Australia; ^4^ Department of Marketing, Faculty of Business and Economics University of Antwerp Antwerp Belgium; ^5^ Cancer Council Western Australia Subiaco WA Australia; ^6^ Mental Health Commission Perth WA Australia; ^7^ School of Medical and Health Sciences Edith Cowan University Joondalup WA Australia; ^8^ Nutrition & Health Innovation Research Institute Edith Cowan University Joondalup WA Australia; ^9^ School of Medical and Health Sciences Edith Cowan University Bunbury WA Australia

**Keywords:** Australia, children, fast‐food, obesity, schools, socio‐economic status

## Abstract

**Issue addressed:**

Locating fast‐food outlets near schools is a potential public health risk to schoolchildren, given the easy access and repeated exposure to energy‐dense, nutrient‐poor foods they provide. Fast‐food outlet availability near schools has not been previously investigated in Perth, Western Australia. This study aimed to quantify fast‐food outlet availability near Perth schools and determine whether differences in area‐level disadvantage and school type exist.

**Methods:**

Fast‐food outlet locations were sourced from Perth Local Governments in 2018/2019. All Perth Primary (n = 454), Secondary (n = 107) and K‐12 (n = 94) schools were assigned an area‐level disadvantage decile ranking based on the Australian Bureau of Statistics Socio‐Economic Index for Areas (SEIFA). Regression models assessed whether fast‐food outlet availability within 400 m, 800 m and 1 km of schools differed by school type (ie, Primary/Secondary/K‐12) or SEIFA.

**Results:**

Secondary schools were significantly more likely than Primary and K‐12 schools to have a higher presence and density of fast‐food outlets and the “Top 4” fast‐food outlet chains (McDonalds, Hungry Jacks, KFC and Red Rooster) nearby. Schools located in low socio‐economic status (SES) areas had a significantly higher density of fast‐food outlets within 400 m, and “Top 4” fast‐food outlet chains within 400 m and 1 km, than schools located in high SES area.

**Conclusions:**

Perth schools are surrounded by fast‐food outlets with densities significantly higher around secondary schools and schools located in lower SES areas.

**So what?:**

Policies and regulations aimed at reducing fast‐food outlets near schools is an essential strategy to improve dietary intakes and reduce obesity in schoolchildren.

## 1 INTRODUCTION

One in three Australian children are overweight or obese and the majority do not consume a diet consistent with Dietary Guidelines.^1^ A major contributor to childhood overweight and obesity is the frequent intake of “discretionary foods” (ie, foods high in energy but low in nutritional value, such as fast‐food and soft‐drink). On any typical day in Australia, half of all teenagers drink soft‐drink and one quarter consume a burger and/or chips.^1^ These types of foods often displace core nutritious foods such as vegetables, fruit and dairy from children's diets.^2^ Less than 10% of Australian children meet the recommended daily intake for vegetables^1^ and for Western Australian (WA) schoolchildren, approximately 38% of total daily energy intakes are obtained from “discretionary foods.”^3^

Having unhealthy food environments near schools can adversely affect diet quality and be a driver of obesity in schoolchildren. A recent WA study found almost half (45%) of secondary students purchased discretionary foods from food outlets near their school on a weekly‐or‐more basis^4^; furthermore, purchase frequency was significantly associated with the availability of major fast‐food outlet (FFO) chains (McDonald's™, Hungry Jack's™, Red Rooster™, KFC™) near the school.^4^ International studies also show many children visit food retailers on their way to/from school, mostly purchasing discretionary foods^5^, ^6^, ^7^, ^8^, ^9^, ^10^; and that schools in deprived areas are surrounded by higher densities of FFO's and convenience stores than schools in less deprived areas.^11^, ^12^ This suggests socio‐economically disadvantaged school populations may be at heightened risk of developing poor eating habits due to increased exposure to unhealthy foods. In Australia, 38% of secondary schools in Victoria were shown to have a FFO within 1 km^13^ and in Adelaide (South Australia), socio‐economically disadvantaged schools had significantly higher densities of FFO's nearby than schools located in socio‐economically advantaged areas.^14^ The availability of FFO's near WA schools is yet to be investigated.

Current State priorities (eg, the WA Sustainable Health Review) identify the need to promote and foster healthy eating environments,^15^ specifying “Changes to planning laws to limit unhealthy food outlets and to support access to healthy food options, including near schools*”* as a priority for implementation. This priority is laudable, yet there is no detailed local evidence to benchmark and guide implementation. Therefore, the aim of this study was to quantify the availability of FFO around all Perth metropolitan schools in WA and investigate whether differences in area‐level disadvantage and school type exist.

## 2 MATERIALS AND METHODS

A listing of all schools (n = 655), including primary (n = 454, 69.3%), secondary (n = 107, 16.3%) and K‐12 (n = 94, 14.4%) located within the Perth metropolitan area in 2019 was obtained from the WA Education Department and geocoded (ie, mapped in ArcGIS v10.6). The Perth metropolitan area was selected as it is the largest city within WA, with the selected sample size representing about 58% of all schools (public and private) in WA (n = 1,139).^16^ All schools were allocated a suburb‐level disadvantage decile ranking based on the Australian Bureau of Statistics Socio‐Economic Index for Areas (SEIFA) Index of Relative Socio‐Economic Disadvantage.^17^ Decile 1 indicates suburbs with relatively greater disadvantage (eg, many people with no qualifications or low skilled occupations, less car ownership), whereas decile 10 indicates a relative lack of disadvantage (eg, few people with no qualifications or low skilled occupations, greater car ownership).

The locations of FFO's were sourced from each Perth Local Government Between May 2018 and July 2019. FFO's were defined as a food outlet where food is ordered at the counter, served immediately and can be eaten without cutlery (eg, burger, ice‐cream, donut and pizza shops). A separate sub‐variable was created to distinguish the “Top 4” most frequented FFO chains (ie, McDonalds™, KFC™, Hungry Jacks™ and Red Rooster™, based on market research^18^) in Australia. Counts (density) of FFO's and “Top 4” FFO chains within Euclidian (circular) buffers of 400 m, 800 m and 1 km around schools were calculated. Logistic binomial regression assessed whether FFO and “Top 4” FFO chain availability differed by school type (ie, Primary/Secondary/K‐12). Negative binomial or Poisson regression was used to calculate incidence rate ratios to assess whether FFO and “Top 4” FFO chain availability around schools differed by school SES (low vs high).

## 3 RESULTS

Table 1 displays the availability of FFO's and “Top 4” FFO chains near schools in Perth, WA by school type. Ninety‐seven percent of secondary schools had one or more FFO within 1 km, with an average of 12.6 FFO's per school. About half of secondary schools (51%) had access to more than one “Top 4” FFO chains within 1 km, which was higher than primary (39%) and K‐12 schools (36%). Reducing the Euclidian buffer meant fewer FFO's present, however, almost half of all schools still had FFO's present within 400 m. Secondary schools were significantly more likely than primary and K‐12 schools to have more FFO's within 400 m, 800 m and 1 km, and more “Top 4” FFO chains within 800 m and 1 km. Secondary schools were significantly more likely than primary and K‐12 schools to have at least one FFO or “Top 4” FFO chain within 1 km. Secondary schools were also significantly more likely than K‐12 schools to have at least one FFO within 800 m. There were 174 schools (26.6%) located in low SES areas (SEIFA deciles 1‐3) and 254 schools (38.8%) located in high SES areas (SEIFA deciles 8‐10). Table 2 displays the availability of FFO's and “Top 4” FFO chains near schools in Perth, WA by area‐level SES decile ranking of the school. Schools located in low SES areas had a significantly higher frequency of FFO's within 400 m, and “Top 4” FFO chains within 400 m and 1 km, compared to schools located in high SES areas.

**TABLE 1:** Availability of fast‐food outlets and “Top 4” fast‐food outlet chains near schools in Perth, Western Australia by school type

	Total outlet count (average per school)					Logistic binomial regression results		
	Euclidian buffer around schools	Primary	Secondary	K‐12	All schools combined	Primary relative to K‐12	Primary relative to secondary	K‐12 relative to secondary
Total count of fast‐food outlets	400 m	724 (1.6)	317 (3)	159 (1.7)	1200 (1.8)	0.94 (0.60‐1.47)	**0.54 (0.36‐0.81)** **	**0.57 (0.33‐0.99)** *
	800 m	2282 (5)	982 (9.2)	480 (5.1)	3744 (5.7)	0.98 (0.74‐1.32)	**0.55 (0.42‐0.72)** ***	**0.56 (0.39‐0.80)** **
	1 km	3261 (7.2)	1351 (12.6)	670 (7.1)	5282 (8.1)	1.01 (0.78‐1.29)	**0.57 (0.45‐0.72)** ***	**0.56 (0.41‐0.77)** ***
Total count of “Top 4” fast‐food chains	400 m	71 (0.2)	30 (0.3)	12 (0.1)	113 (0.2)	1.23 (0.54‐2.80)	0.56 (0.28‐1.10)	0.46 (0.17‐1.19)
	800 m	242 (0.5)	82 (0.8)	42 (0.4)	366 (0.6)	1.19 (0.78‐1.83)	**0.70 (0.48‐1.00)** *	**0.58 (0.35‐0.97)** *
	1 km	287 (0.6)	99 (0.9)	44 (0.5)	430 (0.7)	1.35 (0.94‐1.94)	**0.68 (0.52‐0.91)** **	**0.51 (0.33‐0.77)** **

	Total school count (%)					Logistic binomial regression results		
	Euclidian buffer around schools	Primary	Secondary	K‐12	All schools combined	Primary relative to K‐12	Primary relative to secondary	K‐12 relative to secondary
≥1 fast‐food outlet is present	400 m	199 (43.8)	52 (48.6)	40 (42.6)	291 (44.4)	0.05 (0.23)	−0.19 (0.22)	−0.24 (0.28)
	800 m	352 (77.5)	92 (86.0)	64 (68.1)	508 (77.5)	0.48 (0.25)	−0.58 (0.30)	**−1.06 (0.36)** **
	1 km	389 (85.7)	104 (97.2)	76 (80.9)	569 (86.9)	0.35 (0.29)	**−1.76 (0.60)** **	**−2.11 (0.64)** **
≥1 “Top 4” fast‐food chain is present	400 m	46 (10.1)	15 (14.0)	10 (10.6)	71 (10.8)	−0.05 (0.37)	−0.37 (0.32)	−0.31 (0.44)
	800 m	142 (31.3)	43 (40.2)	27 (28.7)	212 (32.4)	0.12 (0.25)	−0.39 (0.22)	−0.51 (0.30)
	1 km	179 (39.4)	55 (51.4)	34 (36.2)	268 (40.9)	0.14 (0.24)	**−0.49 (0.22)** *	**−0.62 (0.29)** *

*Note*: Statistically significant results in bold.

* *P* = 0.05;

** *P* = 0.01;

*** *P* = 0.001.

**TABLE 2:** Availability of fast‐food outlets and “Top 4” fast‐food outlet chains near schools in Perth, Western Australia by area‐level socio‐economic status (SES) decile ranking of the school

	Total outlet count (average)			Incident rate ratio
	Euclidian buffer around schools	Low SES	High SES	Low SES relative to high
Fast‐food outlet	400 m	276 (1.6)	412 (1.6)	**1.32 (1.02‐1.71)** *
	800 m	1060 (6.1)	1171 (4.6)	0.98 (0.66‐1.45)
	1 km	1406 (8.1)	1769 (7.0)	1.16 (0.93‐1.44)
“Top 4” fast‐food chains	400 m	28 (0.2)	29 (0.1)	**1.86 (1.30‐2.65)** ***
	800 m	122 (0.7)	96 (0.4)	1.41 (0.70‐2.85)
	1 km	137 (0.8)	122 (0.5)	**1.64 (1.23‐2.18)** ***

*Note*: Statistically significant results in bold.

* *P* = 0.05;

*** *P* = 0.001.

## 4 DISCUSSION

This study is the first to quantify FFO availability around schools in Perth, WA. Overall, we found Perth schools are surrounded by FFO's. Around 87% of all Perth schools had a FFO present within 1 km; with nearly half (44%) having a FFO within just 400 m. One in 7 Perth secondary schools (14%) had at least one of the “Top 4” FFO chains within just 400 m (less‐than a 5‐minute walk). This is consistent with a prior Australian study finding 13% of Victorian secondary schools had at least one “Top 4” FFO chain within 500 m.^13^ Students are likely to still access these proximal outlets before and after school regardless of their mode of travel to/from school (ie, driven, public transport or active travel). These findings are concerning given Australian and International research has found exposure to fast‐food outlets around schools increases unhealthy dietary intake^19^ and unhealthy food purchases^4^ in adolescents.

Studies examining trends in retail mix over‐time suggest the number of unhealthy food outlets near schools are increasing. For example, in the United Kingdom, the number of convenience stores within 800 m of schools increased significantly between 2001 and 2005^19^; and in New Zealand, the median number of supermarkets within 800 m of schools decreased from 5 to 1, while the median number of FFO increased from 1 to 4, between 1966 and 2006.^20^ Future research should investigate whether the relative density of unhealthy food outlets near schools is increasing in Australia. Furthermore, the overall mix of food outlets around schools may have dietary implications by influencing healthy and unhealthy food choices. Thus, further investigation of the associations between food purchasing behaviour, dietary intake and the mix of food outlets around Perth schools is required.

Our study found differences in the availability of FFO's near schools by school type; secondary schools were significantly more likely than primary and K‐12 schools to have a higher density and presence of FFO's and “Top 4” FFO chains nearby. It is plausible that FFO's choose to be sited near secondary schools as strategically it makes good business sense; secondary students have more autonomy over food choices, more disposable income and are more independently mobile compared with primary‐school aged children. They are also among the biggest consumers of fast‐food.^1^

Our study also found significantly greater FFO availability around schools located in low (vs high) SES areas. Similar findings have been reported in Adelaide (South Australia),^14^ Victoria (Australia),^13^ New Zealand and the United States.^11^, ^12^ Greater densities of FFO's in low SES areas may reflect lower land costs and thus greater financial gains for food outlet owners. Alternatively, it could be an issue of demand, with business owners wanting to target low SES areas as this is where fast‐food intake is highest.^21^ Nevertheless, a socio‐economic disparity in FFO availability near schools clearly exists in Australia, suggesting these vulnerable populations are at heightened risk of developing poor eating habits as a result of this increased exposure to unhealthy foods.

Overall, our results suggest that policies and regulation aimed at reducing the availability of FFO near schools are essential to improve dietary intakes and reduce obesity in WA schoolchildren. Internationally, efforts have been made to limit schoolchildren's exposure to unhealthy food outlets through urban planning measures. For example, the United Kingdom, the United States, Ireland and South Korea have introduced restrictions on the locations of FFO's around schools. While not specific to schools, in 2008 the Los Angeles City Council approved a 1‐year moratorium on the opening of new FFO's in low‐income neighbourhoods with an already high FFO density and in 2011 extended the moratorium indefinitely.^22^ Furthermore, in Gateshead council within Northeast England, there has been a reported decrease in the Density and proportion of FFO following the restriction of new FFO.^23^ However, research is yet to investigate the downstream impacts of planning and land‐use restrictions. It may be that using planning to change dietary intakes is a long‐term policy plan that starts by changing the local food environment.

By comparison, Australia is lagging behind with no present examples of fast‐food zoning restriction or regulations near schools. Currently, in WA, “public health” is not deemed a relevant planning consideration. The *Planning and Development (Local Planning Schemes) Regulations 2015 (WA)* provides a set of regulations that govern the way local planning strategies and local planning schemes are prepared and amended. Regulation 67 of Schedule 2 of the *Planning and Development (Local Planning Schemes) Regulations 2015 (WA)* relates to the consideration and approval of development applications by local Government. If Regulation 67 of Schedule 2 of the *Planning and Development (Local Planning Schemes) Regulations 2015 (WA)* was amended to include public health considerations, then Local Governments would have additional provisions over the future installation of FFO's and the health and wellbeing of their residents. For example, Local Governments would be able to influence land use planning policies in school catchment areas or introduce exclusion/restriction zones with prescribed minimum distances for FFO's located near schools based on health grounds. This is also the case for most other states (with the exception of Queensland and Tasmania), where overarching planning laws do not allow for preventative health considerations to impact planning decisions.^24^ Therefore, the findings from this study have policy implications across Australia.

Restricting FFO's around schools was identified as a key issue by members of a citizens jury.^25^ In WA, there is growing community concern regarding the “obesogenic” environment around schools. For example, Warnbro Community High School was forced to build a metal fence to stop students sneaking out to nearby FFO's.^26^ Several other Perth secondary schools have had to ask local FFO's to stop serving their students during school hours.^27^ Many of these schools have FFO's so close by they are visible from classroom windows.^28^ Recent calls from the community for a ban on FFO's near schools,^26^ highlight the need for regulation to address the location of FFO's near schools, while at the same time, ensuring school canteens offer food that is affordable, healthy and attractive to students.

Limitations of this study include its cross‐sectional design (ie, it assessed FFO availability at only one point in time) and focus on a single Australian city.^1^ However, the study scored well on the BMJ's appraisal tool for cross‐sectional studies (AXIS) (Supplementary File 1).^29^ It may be the case that secondary schools and schools in low SES areas are located where there is an overall greater density of all food outlets, including healthy food outlets. Furthermore, this study did not account for population density, which can also influence the density of FFO. Future research should examine all food outlet types (eg, supermarkets, convenience stores, etc) and assess trends in retail mix over time across all Australian states and territories. Lastly, this study did not quantify the healthiness of food sold within the examined fast‐food outlets and it may be possible that some fast‐food outlets also sell healthy food. This study used reliable and valid measures of FFO availability and has provided much needed insights and benchmarking data to guide the implementation of future policies aiming to improve the healthfulness of food environments surrounding schools locally and outside Australia.

## FUNDING INFORMATION

This study was funded by a Healthway Research Exploratory Grant (#32981) and Cancer Council Western Australia and the Telethon Kids Institute as part of their Rapid Obesity Policy Translation Program. Dr Trapp is supported by an Australian Research Council DECRA Fellowship (DE210101791). Dr Hooper is supported by a Healthway Research Fellowship (#32892).

## CONFLICT OF INTEREST

The authors declare no conflict of interest.

## ETHICS STATEMENT

This study did not involve research on humans, thus human research ethics approval was not necessary for this research. The University of Western Australia Human Research Ethics Committee (HREC) deemed this study as exempt from HREC review (reference number: 2019/RA/4/1/6524).

## Supporting information


**Supplementary File 1**. Appraisal tool for cross‐sectional studies (AXIS).

## Data Availability

The data that support the findings of this study are available from the corresponding author upon reasonable request.
