# Supplementary material for: Would you like fries with that? Investigating fast‐food outlet availability near schools in Perth, Western Australia
Source: Health Promot J Austr. 2022 Dec 4;34(1):85–90. doi: 10.1002/hpja.682 (PMC10108019; doi:10.1002/hpja.682)
Supplement: Supplementary file 1 — Appraisal tool for cross‐sectional studies (AXIS). [file HPJA-34-85-s001.pdf]

Appraisal of Cross-sectional Studies

	Question	Yes	No	Don't know/ Comment
Introduction				
1	Were the aims/objectives of the study clear?	Yes		
Methods				
2	Was the study design appropriate for the stated aim(s)?	Yes		
3	Was the sample size justified?	Yes		
4	Was the target/reference population clearly defined? (Is it clear who the research was about?)	Yes		
5	Was the sample frame taken from an appropriate population base so that it closely represented the target/reference population under investigation?	Yes		
6	Was the selection process likely to select subjects/participants that were representative of the target/reference population under investigation?	Yes		
7	Were measures undertaken to address and categorise non-responders?			NA
8	Were the risk factor and outcome variables measured appropriate to the aims of the study?	Yes		
9	Were the risk factor and outcome variables measured correctly using instruments/measurements that had been trialled, piloted or published previously?	Yes		
10	Is it clear what was used to determined statistical significance and/or precision estimates? (e.g. p-values, confidence intervals)	Yes		
11	Were the methods (including statistical methods) sufficiently described to enable them to be repeated?	Yes		
Results				
12	Were the basic data adequately described?	Yes		
13	Does the response rate raise concerns about non-response bias?			NA
14	If appropriate, was information about non-responders described?			NA
15	Were the results internally consistent?	Yes		
16	Were the results presented for all the analyses described in the methods?	Yes		
Discussion				
17	Were the authors' discussions and conclusions justified by the results?	Yes		
18	Were the limitations of the study discussed?	Yes		
Other				
19	Were there any funding sources or conflicts of interest that may affect the authors' interpretation of the results?		No	
20	Was ethical approval or consent of participants attained?			NA
